# Massive neurocysticercosis in a ten-year-old girl: a case report

**DOI:** 10.1186/s12887-024-04530-7

**Published:** 2024-01-24

**Authors:** Guoguang Xiao, Min Shu

**Affiliations:** 1 Department of Pediatrics, West China Second Hospital, Sichuan University, No. 20, Section 3, Renmin South Road, Chengdu, 610041 Sichuan Province P. R. China; 2 Key Laboratory of Birth Defects and Related Diseases of Women and Children (Sichuan University), Ministry of Education, Chengdu, 610041 China; 3 West China Xiamen Hospital of Sichuan University, Xiamen, 361022 China

**Keywords:** Massive neurocysticercosis, Neuroimaging, Antiparasitic treatment, Case report, Child

## Abstract

**Background:**

Massive neurocysticercosis is a rare form of neurocysticercosis, and can lead to serious conditions and even death.

**Case presentation:**

Here we present a case of ten-year-old Tibetan girl who developed headache and vomiting. Her brain magnetic resonance imaging (MRI) illustrated lots of intracranial cystic lesions, and no obvious extracranial lesions were found. Serum immunoglobulin G antibodies against cysticerci were positive by the use of an enzyme-linked immunosorbent assay (ELISA). These results in combination with her medical history were in line with massive neurocysticercosis. The patients recovered well after supportive management and antiparasitic treatment.

**Conclusions:**

This case provides insights on the diagnosis and treatment of massive neurocysticercosis. The treatment of patients with massive neurocysticercosis should be in an individualized fashion, and the use of antiparasitic drugs in these patients must be decided after carefully weighing the risks and benefits.

## Background

Neurocysticercosis is an important neurologic infectious disease which results from the infection of cystic larvae of *Taenia solium* through fecal-oral route. It has been reported that neurocysticercosis is an important cause of seizure disorders [1, 2]. Massive neurocysticercosis is a rare form of neurocysticercosis, and can lead to serious conditions and even death. In the English literatures, reports of massive neurocysticercosis have been published in only a few case reports or case series until recently, the majority of which are adults and from India, and lots of them have been published in the section of Clinical Image within medical journals [3, 4]. Detailed discussions on massive neurocysticercosis have been scarce until recently. Few cases have been reported in China, especially in the child population. Here we present a case of child massive neurocysticercosis with satisfactory treatment effect and propose a brief review on some important aspects of this disease.

## Case presentation

A 10-year-old Tibetan girl presented with headache and nonprojectile vomiting for two months. Her headache was moderate to severe in intensity. No fever, seizure, or weight loss was reported. The girl had no significant medical history previously, and her family members were in good health. Her six schoolmates presented with similar symptoms and had been suspected of cysticercosis. Cooked yak meat and pork were the main source of meat for the patient and her family.

**Fig. 1:**
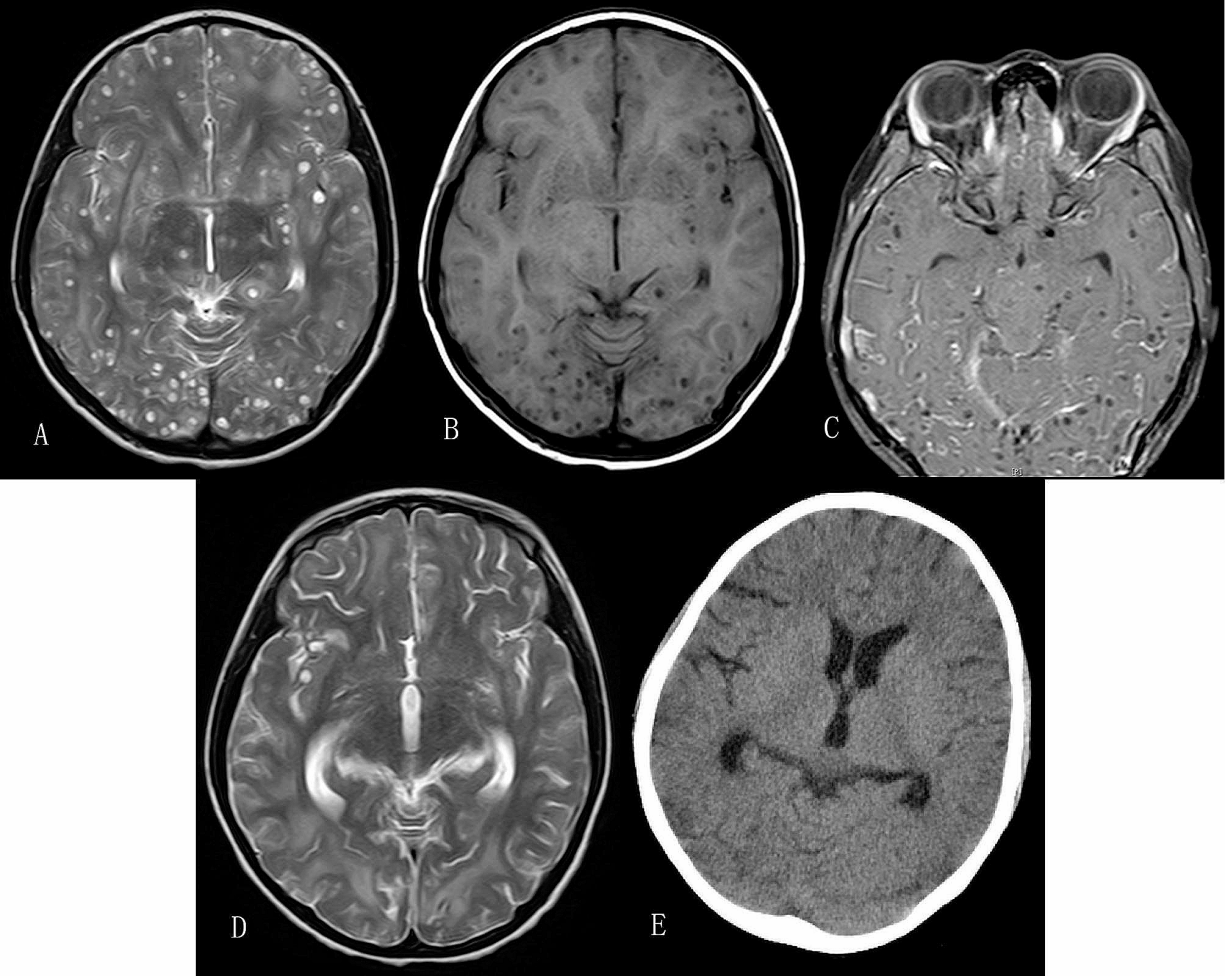
MRI findings of intracranial lesions at baseline and follow-up assessments. Axial T2-weighted (**A**) and T1-weighted (**B**) magnetic resonance images before antiparasitic treatment showed massive cystic lesions with the so-called “hole-with-dot” appearance, indicating viable cysticercus with eccentric scolex, which has been regarded as pathognomonic characteristic of neurocysticercosis. **C**: Contrast-enhanced MRI image of the eyes of the patient which also showed some intracranial lesions before antiparasitic treatment. **D**: Brain axial T2-weighted MRI image two months after antiparasitic treatment demonstrated resolution of most intracranial cystic lesions. **E**: Brain CT scan six months after antiparasitic therapy showed very few residual calcifications

At admission, general and neurological examinations were unremarkable. Blood routine tests showed that white blood cell count was 7.5 × 10^9^/L with a percentage of eosinophil of 7.8% (normal range, no more than 6.9%), an absolute eosinophil count of 0.58 × 10^9^/L (normal range, no more than 0.68 × 10^9^/L), and normal absolute neutrophil and lymphocyte count. Examination of cerebrospinal fluid (CSF) showed slight elevation of protein concentration (696.4 mg/L; normal range, 80-430 mg/L) and white blood cell count (25 × 10^6^/L; normal range, less than 15 × 10^6^/L), and normal glucose. Gram and Indian ink stains and bacterial culture of CSF were negative. C-reactive protein, serum immunoglobulin E, serology for human immunodeficiency virus and syphilis, T-SPOT.TB (an enzyme-linked immunospot assay for the infection of tuberculosis), tuberculin skin test, stool examination, abdominal ultrasound, chest computed tomography (CT), ocular and spinal magnetic resonance imaging (MRI) revealed no positive results. Brain MRI showed a lot of cystic lesions and somewhat perilesional edema in cerebral hemispheres, brain stem and cerebellum, and some lesions presented with the so-called “hole-with-dot” appearance, indicating viable cysticerci with eccentric scolex (Fig. 1A-C). Serum immunoglobulin G antibodies against cysticerci were detected using an enzyme-linked immunosorbent assay (ELISA). These results were consistent with a diagnosis of massive parenchymal neurocysticercosis.

Corticosteroids and mannitol were administered to decrease brain edema and inflammation. Albendazole (15 mg/kg/d for 10 days) was given to the patient, and her symptoms were relieved. However, headache re-emerged after the treatment was over for one month, accompanied by an episode of seizure and intermittent arm and leg shaking movements. The shaking movements lasted several minutes to half an hour each time without impaired awareness, and happened mainly in the morning with an interval of several days. Repeated brain MRI showed no significant change of these intracranial lesions. Then supportive management including corticosteroids and mannitol and retreatment of albendazole (15 mg/kg/d) for 10 days were given to the patient. Oral prednisone was continued after these treatments. Headache and shaking movements of limbs were relieved. About three weeks after the end of these treatments, headache and an episode of seizure recurred. Considering that the patients did not respond very well to the therapeutic regimen of albendazol alone, albendazol (15 mg/kg/d) in combination with praziquantel (50 mg/kg/d) was administered for 14 days. Oral prednisone was given at the same time and in additional two weeks after the antiparasitic therapy. The patient’s symptoms were all relieved. Repeated imaging two months after antiparasitic treatment showed that most cystic lesions had resolved (Fig. 1D). The patient recovered well within the 4-year follow-up. The good therapeutic response to the antiparasitic medication also supported our diagnosis in this patient.

## Discussion and conclusions

Neurocysticercosis, the most common helminthic infection of the nervous system, is caused by the infection of cystic larvae of *T. solium*. It is still endemic in many low-income countries such as Latin America, sub-Saharan Africa, Southeast Asia and some parts of China, and has been increasingly diagnosed in developed countries nowadays due to immigration and international travel [5].

Two hosts are involved in the life cycle of *T. solium*, with pigs acting as an intermediate host and humans as both definitive and intermediate hosts. Human cysticercosis results from ingestion of eggs of *T. solium* mainly by fecal-oral route. Eggs in human digestive tract can evolve into oncospheres which can migrate through the bloodstream into the central nervous system leading to neurocysticercosis and into other tissues resulting in other types of human cysticercosis.

Massive neurocysticercosis is scarce and sometimes with very poor outcome [3]. Studies have reported that the Toll-like receptor-4 gene polymorphisms may be responsible for the increased incidence of disseminated cysticercosis including massive neurocysticercosis among the Indian population [6]. However, this needs to be verified in larger studies. In addition, the disease in the early phase has nonspecific symptoms and sometimes is asymptomatic.

Clinical presentations of neurocysticercosis are pleomorphic and depend on the number and viability of cysts, location such as within the brain parenchymal, subarachnoid space, ventricular system and spinal cord, as well as the intensity of host immunological response. The most common clinical presentations of neurocysticercosis include seizures, headache and vomiting, focal neurological deficits, and cognitive alterations. Parenchymal neurocysticercosis is the most common form and represents more than 60% cases [7]. Extraparenchymal neurocysticercosis includes ventricular, subarachnoid, spinal, and ocular neurocysticercosis. Patients with massive parenchymal neurocysticercosis could have evident inflammatory reaction to the cysticerci and diffused cerebral edema, and present with severe headaches and altered consciousness, which is denominated “cysticercotic encephalitis”, the most severe form of massive parenchymal neurocysticercosis. Other forms of massive parenchymal neurocysticercosis can be described as massive parenchymal calcifications, heavy nonencephalitic cysticercosis, and starry sky presentation [7]. Our patient presented no typical manifestations of cysticercotic encephalitis and had been regarded as “heavy nonencephalitic neurocysticercosis”. It is interesting that no obvious extracranial cysticercosis lesions were found in our patient, which is different from most other reports in which massive parenchymal neurocysticercosis often accompanied by extracranial lesions called disseminated cysticercosis [3, 4].

The diagnosis of neurocysticercosis should be based on neuroimaging as well as immunodiagnostic tests, and clinical presentations and epidemiological data could provide supportive information [8, 9]. Despite being the gold standard for the diagnosis of neurocysticercosis, histological confirmation of the parasite is not possible in most cases. Response to drug treatment is also useful for making the diagnosis, as shown in our case [8]. Neuroimaging is essential for the diagnosis of neurocysticercosis. In the brain parenchyma, cysticerci can exhibit different characteristics on neuroimaging examinations according to their stage, which include: well-defined cystic lesions without perilesional edema and enhancement (vesicular cysticerci), cystic or nodular lesions presenting with perilesional enhancement after contrast medium administration (colloidal and granular cysticerci), and small calcifications (calcified cysticerci) [10]. The presence of vesicular cysts with eccentric scolex on neuroimaging has been regarded as the pathognomonic appearance of neurocysticercosis [8, 9]. Specific serologic assays could give supportive information for the diagnosis of neurocysticercosis. The enzyme-linked immunotransfer blot (EITB) using lentil lectin purified glycoprotein antigens is recommended, and is superior to the ELISA for identification of anticysticercal antibodies in serum [10]. However, the technical complexity of EITB limits its widespread availability. ELISA is still used in many areas where EITB is not available. The results of ELISA should be interpreted carefully for the relatively low specificity and sensitivity of this method. Recently an easy cyst fluid based EITB has been reported, and the results showed that certain reactive antigen band based on this method was highly sensitive and specific for the diagnosis of neurocysticercosis including single cyst infection among endemic populations [11]. Molecular methods including quantitative real-time polymerase chain reaction (qPCR) assay and metagenomic next-generation sequencing have been reported in some studies [12, 13].

Differential diagnoses of this disease include other intracranial infections such as tuberculomas, brain abscesses, and sometimes other parasitic infections (for example, *Echinococcus granulosus*, *Paragonimus* species), and tumors like metastases also should be considered [9].

Therapeutic regimen for treating neurocysticercosis must be tailored according to the specific clinical manifestation of each patient. Symptomatic treatment to control the elevated intracranial pressure and seizures must always be the first line of management. Antiparasitic drugs including albendazole and praziquantel have improved the prognosis of many patients diagnosed with neurocysticercosis and play an important role in the management of some types of this disease. Nevertheless, because antiparasitic drugs could exacerbate the inflammatory reaction which can worsen symptoms in the short term, these drugs should be given under the cover of anti-inflammatory drugs such as corticosteroids [7]. Antiparasitic therapy is contraindicated in patients with cysticercotic encephalitis. Some reports indicate that cases with heavy nonencephalitic neurocysticercosis may benefit from the treatment of corticosteroids and antiparasitic medication [6]. After carefully weighing the risks and benefits, antiparasitic therapy in combination with corticosteroids and other symptomatic management was given to our patient. Additionally, surgery may be needed in some cases.

As mentioned above, neurocysticercosis is regarded as among the most common parasitic brain infections associated with seizure disorders worldwide. Brain calcified lesion resulting from cyst degeneration can be permanent focus of seizures and other neurological symptoms. Studies have suggested that some factors including shorter duration of epilepsy, less cystic lesions at the time of diagnosis, antiparasitic therapy especially combination therapy using albendazole together with praziquantel, and higher doses of corticosteroids seemed to be associated with lower risk of residual calcification [14, 15]. Although with heavy parasite burden, our patient responded well to therapeutic regimen of albendazole combined with praziquantel with very few residual calcifications as shown in the follow-up brain CT scan conducted six months after antiparasitic treatment (Fig. 1E). As one study reported that higher rates of calcifications occurred at later time points, for example, at two years (vs. one year) after antiparasitic therapy [15], a limitation of our case is the lack of brain CT scans at the time points of one year and two years after antiparasitic treatment to further investigate the formation of calcified lesions.

Furthermore, it has been reported that differences in infection pressure alter the incidence/prevalence of the different types of cysticercosis. Here infection pressure determines the risk of a person ingesting the eggs of *T. solium* [16]. A recent study from South Korea showed that in the context of a decreasing overall neurocysticercosis incidence in this country due to the decrease of infection pressure, the incidence of parenchymal neurocysticercosis decreased during the period of 2000–2016 compared with the period of 1990–1999, and the incidence of extraparenchymal neurocysticercosis increased between the two periods [17]. Under circumstance of high infection pressure, it seems that parenchymal neurocysticercosis is the most frequent type of neurocysticercosis. The infection pressure might be high in the region where this patient came from for the reason that this patient was a case of massive parenchymal neurocysticercosis and six other schoolmates had been suspected of cysticercosis. However, epidemiological investigations on infection of *T. solium* within this region have been scarce until recently. Therefore, epidemiological study of the prevalence of cysticercosis in this area would be the next work to be done.

*T. solium* has been regarded as a potentially eradicable pathogen [18]. Traditional efforts for disease control including improving sanitation, health education, and inspection of pork were largely unsuccessful in highly endemic areas [7]. Mass chemotherapy for the control of infection of *T. solium* using albendazole and praziquantel has been implemented in some endemic communities. Nevertheless, there have been concerns about insufficient public participation and increasing drug resistance for mass administration of antiparasitic drugs. Vaccination is recognized to be an effective prophylactic measure against infectious diseases. Some veterinary vaccines such as TSOL18 and S3Pvac have been used against pig cysticercosis [19, 20]. However, no vaccine for human neurocysticercosis is available. There has been certain progress in identifying the immune reactive proteins to help the development of vaccines against *T. solium*, and some novel research methods have been used in these studies [21]. Further studies on the development of vaccine against human cysticercosis are badly needed.

In conclusion, the diagnosis of neurocysticercosis is complicated and mainly depends on neuroimaging findings together with immunodiagnostic tests. Careful epidemiological data taking and response to treatment could also provide useful information. Individualized treatment regimens should be given to patients with different types of neurocysticercosis. The use of antiparasitic drugs in patients with massive neurocysticercosis must be decided after carefully weighing the risks and benefits.

## Data Availability

The data supporting the findings of this study are available from the corresponding author upon reasonable request.

## Abbreviations

CSF: Cerebrospinal fluid
CT: Computed tomography
MRI: Magnetic resonance imaging
ELISA: Enzyme-linked immunosorbent assay
EITB: Enzyme-linked immunotransfer blot
qPCR: Quantitative real-time polymerase chain reaction
